# Theoretical Insights into the Structures and Capacitive Performances of Confined Ionic Liquids

**DOI:** 10.3390/polym12030722

**Published:** 2020-03-24

**Authors:** Jie Yang, Yajun Ding, Cheng Lian, Sanjiu Ying, Honglai Liu

**Affiliations:** 1 School of Chemical Engineering, Nanjing University of Science and Technology, Nanjing 210094, China; 2 State Key Laboratory of Chemical Engineering, Shanghai Engineering Research Center of Hierarchical Nanomaterials, School of Chemistry and Molecular Engineering, East China University of Science and Technology, Shanghai 200237, China

**Keywords:** supercapacitors, ionic liquids, classical DFT, microscopic structures

## Abstract

Room-temperature ionic liquids (RTILs) together with nano-porous electrodes are the most promising materials for supercapacitors and batteries. Many theoretical works have addressed the structures and performances of RTILs inside nanopores. However, only limited attention has been given to how the dispersion forces of RTILs influence the behavior of ions inside the slit pores. Toward this aim, we investigate the effects of various dispersion forces between ions on the macroscopic structures in nanoconfinement and the capacitance performance of supercapacitors by the classical density functional theory (CDFT). The results show that the dispersion force can significantly change the mechanism of the charging process and even the shape of differential capacitance curves. In addition, the voltage-dependent structures of RTILs with appropriate dispersion force appears in a given silt pore, which leads to extremely high capacitance and enhances the energy storage density. We hope that this work could further offer guidance for the optimizing of electrolytes for electrical double layer capacitors, like tuning the dispersion force between ions by adding/removing certain chemical groups on the cations and anions of RTILs.

## 1. Introduction

Electrical double layer capacitors (EDLCs), also known as supercapacitors, offer the unique advantages of high-power density and long cycling lifespans but provide moderate energy storage [1,2]. While batteries’ performance is strongly related to the surface reactions, the adsorption of ions inside the micropores of electrodes plays a critical role in the power and energy density of supercapacitors [3,4,5,6,7]. Supercapacitors store a charge by the formation of electrical double layers at the electrode–electrolyte interfaces when applied to an external potential [8]. In order to enhance the energy density without compromising the mentioned superiority, extensive research effort has been devoted into developing novel electrode materials and electrolytes. The combination of electrodes featuring nanometer pores and room-temperature ionic liquids (RTILs) are the most promising solution for the widespread application of next-generation supercapacitors. The porous materials provide a high specific surface area [1,9,10,11], while the RTILs with excellent thermal stability and nonvolatility [12,13,14] allows for a higher operating potential window (OPW) than conventional electrolytes [15], leading to impressive improvements in energy density [4,5,16,17,18,19,20,21,22,23]. Compared with modifying the pore size distribution, morphology, architecture and functionality of electrode materials [10,24,25,26], it is more convenient and economic to improve the capacitive performance by choosing novel RTILs. 

Despite the fact that a variety of RTILs are widely used in EDLCs, our understanding of the behavior of RTILs at charged interfaces remains elusive [27]. Most surging focus is on the role of steric and electrostatic forces in the behavior of ionic liquids [28,29], which are much stronger than that in usual electrolytes. However, the effects of the intermolecular dispersion force on the EDL structures and capacitances have been less explored. It is generally known that RTILs consist of large polarizable molecules at high density, so that the effects of a dispersion force cannot be negligible at interfaces and under confinement which may lead to different charging mechanisms and capacitive performances. Previous studies have used Monte Carlo (MC) [30] and classical density functional theory (CDFT) [31] simulation to understand their effects on the differential capacitance at planar electrolyte. A simple coarse-grained model based on Lennard-Jones monomers showed that the dispersion force plays a critical role in the “camel-shaped” differential capacitance behavior observed experimentally [32,33,34]. Moreover, an extraordinary increase in capacitance was found derived from the thermodynamic response in both RTILs [35,36] and RTILs plus solvent mixtures [37,38,39]. Owing to the significance of dispersion force on EDLC performance, more effort should be devoted to investigate the phase behavior and charging mechanism of RTILs with different dispersion force in nanoconfinement, which will benefit the design of RTILs electrolytes. 

Herein, the effects of dispersion force on the charging mechanism and phase behavior of RTILs in confined nanopores are investigated by CDFT. Increasing studies have shown that CDFT is an ideal tool to study the EDL structure and capacitance of the ionic liquids electrolyte system [40,41,42,43,44,45]. Compared with the all-atom molecular dynamic (MD) simulation, CDFT is much more computationally efficient as CDFT ignores the molecular details. While it neglects atomic details, CDFT can give molecular insights into the electrochemical behavior of RTILs inside nanopores. First, we describe our coarse-grained model for RTILs with dispersion forces and porous electrodes underpinning the CDFT. Next, we discuss the effects of dispersion forces on the surface charge density, the EDL charging mechanism, the capacitance, and the energy density of supercapacitors. In the end, we summarize the main results and enlightenment for possible future experimental work.

## 2. Models and Methods

**Figure 1:**
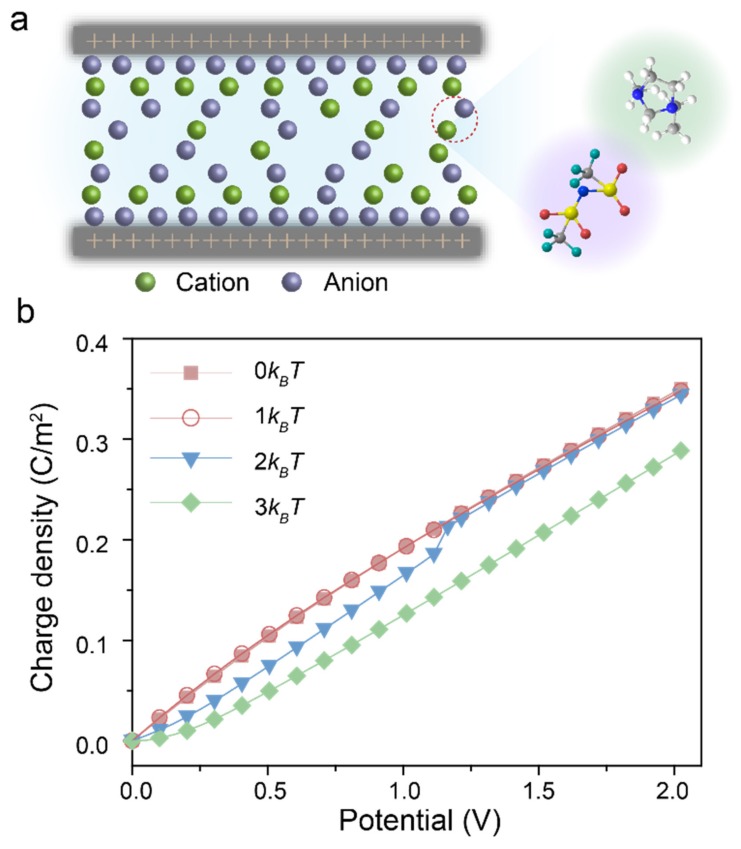
(**a**) Schematic representation of the model used for the classical density functional theory (CDFT) simulation for the 1-ethyl-3-methylimidazolium bis(trifluoromethylsulfonyl) imide (EMIM-TFSI) like room-temperature ionic liquids (RTILs) inside nanopores with dispersion forces between anions and cations. (**b**) Variation of surface charge density versus applied electrical potential of RTILs at different dispersion force $0\;k_{B}T$, $1\;k_{B}T$, $2\;k_{B}T$ and $3\;k_{B}T$, respectively.

Here, a generic model was proposed for EDL capacitors consisting of porous carbons and RTILs. As illustrated in Figure 1a, the electrolyte ions are modeled by the restricted primitive model (RPM), which is a hard sphere with a positive or negative unit electron charge embed at the center. All particles are assumed to have the same size and equal charge. First, we consider the electrochemical potential for ions, in which the dispersion force is seldom taken into account. The electrochemical potential $u_{i}\left(z\right)$ for ions can be divided into two parts as the local chemical potential and the electrostatic component of the external potential respectively, which designated as

(1) $$u_{i}\left(z\right)\equiv \mu_{i}\left(z\right)+V_{i}\left(z\right)$$

where z is the perpendicular distance from the surface, $\mu_{i}\left(z\right)$ denotes the local chemical potential, and $V_{i}\left(z\right)$ is the external potential drawing from the electrodes.

Usually, the electrochemical potential is composed of an ideal solution together with an additional term arising from the mean electric potential, $\psi \left(z\right)$, i.e.,

(2) $$u_{i}\left(z\right)=k_{B}Tln[\rho_{i}\left(z\right)\Lambda_{i}^{3}]+Z_{i}e\psi \left(z\right)$$

where e represents the unit charge, $Z_{i}$ is the ion valence set as $Z_{i}=\pm 1$ and $\Lambda_{i}$ denotes an effective thermal wavelength of ion i. The average electrostatic potential, $\psi \left(z\right)$, is correlated with the local charge density acquired by the Poisson equation,

(3) $$\nabla^{2}\psi \left(z\right)\;=\;-\frac{e}{E_{0}E_{r}}\underset{i}{\sum }Z_{i}\rho_{i}\left(z\right)$$

As reported by previous models of electrolyte solutions [46,47,48,49], besides the ideal solution term and the average electrostatic potential $\psi \left(z\right)$, molecular excluded volume effects, the dispersion forces, and the electrostatic correlations also contribute to the electrochemical potential of each ionic species,

(4) $$u_{i}\left(z\right)=k_{B}Tln[\rho_{i}\left(z\right)\Lambda_{i}^{3}]+eZ_{i}\psi \left(z\right)+\mu_{ex,i}^{HS}\left(z\right)+\mu_{ex,i}^{DIS}\left(z\right)+\mu_{ex,i}^{EC}\left(z\right)$$

where $\mu_{ex,i}^{HS}\left(z\right)$ represents the local excess chemical potential owing to hard sphere repulsion, $\mu_{ex,i}^{DIS}\left(z\right)$ is the contribution of dispersion forces, and $\mu_{ex,i}^{EC}\left(z\right)$ accounts for the eletrostatic correlations. Based on previous work for electrolyte systems at equilibrium, the modified fundamental measure theory (MFMT), the mean-filed approximation and a second-order perturbation theory are applied for $\mu_{ex,i}^{HS}\left(z\right)$, $\mu_{ex,i}^{DIS}\left(z\right)$ and $\mu_{ex,i}^{EC}\left(z\right)$, respectively. 

In accordance with MFMT, the excess chemical potential owing to hard-sphere interactions is expressed by

(5) $$\beta \mu_{ex,i}^{HS}\left(z\right)\begin{array}{c} = \end{array}\begin{array}{c} \underset{\alpha }{\sum }\int dz{}^{\prime }\xi_{\alpha }\left(z{}^{\prime }\right) \end{array}\omega_{i}^{\left(\alpha \right)}\left(z{}^{\prime }-z\right)$$

where $\beta \;=\;1/\left(k_{B}T\right)$, $k_{B}$ is the Boltzmann constant. And the detail expressions for $\xi_{\alpha }$ and $\omega_{i}^{\left(\alpha \right)}$ are given in the previous publications [48,49,50,51,52,53].

As for dispersion interaction between species *i* and *j*, it can be described by square-well potential $u_{ij}^{DIS}(z)=-\varepsilon_{ij}$ for $z<\lambda_{ij}$, where $\lambda_{ij}$ is the range of attraction. We calulate the excess chemical potential arsing from the dispersion attraction approximately by the mean-field approximation.

(6) $$\beta \mu_{ex,i}^{DIS}\left(z\right)=\frac{1}{2}\underset{j=+,-}{\sum }\int \rho_{j}\left(z{}^{\prime }\right)u_{ij}^{DIS}\left(\left|z-z{}^{\prime }\right|\right)dz{}^{\prime }$$

The excess chemical potential owing to the electrostatic correlation is expressed as

(7) $$\beta \mu_{ex,i}^{EC}\left(z\right)=\beta \mu_{b,i}^{EC}-\underset{j=+,-}{\sum }\int \Delta \rho_{j}\left(z{}^{\prime }\right)c_{ij}^{EC}\left(\left|z-z{}^{\prime }\right|\right)dz{}^{\prime }$$

where $\mu_{b,i}^{EC}$ is the excess chemical potential due to the electrostatic correlation of species *i* in the bulk, and $c_{ij}^{EC}\left(z\right)$ represents direct correlation function of bulk fluid owing to electrostatic correlations. The mean-spherical approximation is used to calculate the excess chemical potential contributed from the electrostatic attraction, and details are given in the previous publications [48,49,50,51,52,53].

The porous electrodes were modeled by two symmetric hard walls of equal surface charge density *Q* and separated by the silt pore with width *H*. The non-electrical component of interaction between the silt pore and ion species *i* is modeled as a hard-wall potential *V*_i_ (*z*)

(8) $$V_{i}\left(z\right)\;=\;\{\begin{array}{c} \infty ,\;z\;\leq \;\frac{\sigma_{i}}{2}\;or\;z\;\geq \;H-\;\frac{\sigma_{i}}{2}\\ 0,\;otherwise \end{array}$$

where σ is the ion diameter corresponding to the average size of the ions. We assume that the image-charge effects are relatively unimportant for determining ionic distribution because the residual dielectric constant is similar to the dielectric constant for the carbon electrode. Even at a fixed surface potential, a discontinuity in the dielectric constant at the electrode−electrolyte interface leads to image forces since charge fluctuation near the electrode is correlated with atomic polarization inside the electrode.

Given the bulk ion concentrations, $\rho_{o}^{i}$ and temperature *T*, the ion distributions inside the electrolyte pore *i* could be obtained by CDFT.

(9) $$\rho_{i}\left(z\right)\;=\;\rho_{i}^{0}exp\left[-\beta V_{i}\left(z\right)\;-\;\beta \;Z_{i}e\Psi \left(z\right)\;-\;\beta \Delta \mu_{ex,i}^{HS}\left(z\right)\;-\;\beta \Delta \mu_{ex,i}^{DIS}\left(z\right)\;-\;\beta \Delta \mu_{ex,i}^{EC}\left(z\right)\right]$$

The electrode potential at the surfaces of carbon electrode, $\psi_{0}$, is supposed to be constant such that the boundary conditions for the electrical potential is

$$\psi \left(0\right)=\;\psi \left(H\right)\;=\;\psi_{0}$$

In order to solve Equation (3) and Equation (9) numerically, we assign the bulk concentration as an initial guess of ionic density profiles. Subsequently, the local excess chemical potential and the local electrical potential for each ionic species, $\Delta \mu_{ex,i}^{HS}\left(z\right)$, $\Delta \mu_{ex,i}^{DIS}\left(z\right)$, $\Delta \mu_{ex,i}^{EC}\left(z\right)$ and $\Psi \left(z\right)$, can be calculated from the initial guess and the boundary conditions. Then we replace the values into Equation (9) to acquire a new set of ionic profiles for the next iteration which will stop when the procedure repeats unit convergence ($\left|\Delta \rho_{i}/\rho_{o}^{i}\right|$ < 10^−4^ at all positions). From the ion distributions and the electrical potential profiles, the surface charge density (*Q*) can be readily estimated following the Gauss law:

(10) $$Q\;=\;-\;\underset{i}{\sum }Z_{i}e\int_{0}^{H/2}dz\rho_{i}\left(z\right)$$

## 3. Results and Discussions

Here, the model parameters are selected such that the monomer size and charge approximately match those corresponding to 1-ethyl-3-methylimidazolium bis-(trifluoromethanesulfonyl) imide (EMI-TFSI), a RTIL commonly used in electrochemical devices. The segment diameter of cations or anions is fixed at 0.5 nm. The concentration of RTIL is fixed at 3.85 M, so that the reduced density of bulk ionic liquids is set as $\rho_{b}^{*}=\rho_{+}\sigma^{3}=\rho_{-}\sigma^{3}=0.29$, the dielectric constant $E_{r\;\;}=2,$ and the pore width is H=2.5 nm. The RTILs considered in this theoretical study have similar physical properties so that we can focus on the dispersion force effects on the EDL charging process and capacitance.

Figure 1b shows the CDFT prediction for the surface charge density at four different $\varepsilon_{ij}$, which refers to the dispersion force between cation and anion. The surface charge density of RTILs with strong dispersion force is obviously less than that without dispersion force under applied potential. When the values set as $0\;k_{B}T$ and $1\;k_{B}T$, the charge density increases significantly with the applied potential. Different from the RTILs with weak dispersion forces, the surface charge density of RTILs with stronger dispersion force (e.g., $\varepsilon_{ij}>2\;k_{B}T$) is virtually zero under the surface potential less than 0.2 V, and it increases gradually at a higher potential. Interestingly, there exists a step change as $\varepsilon_{ij}=2\;k_{B}T$ at the potential of 1.16 V.

**Figure 2:**
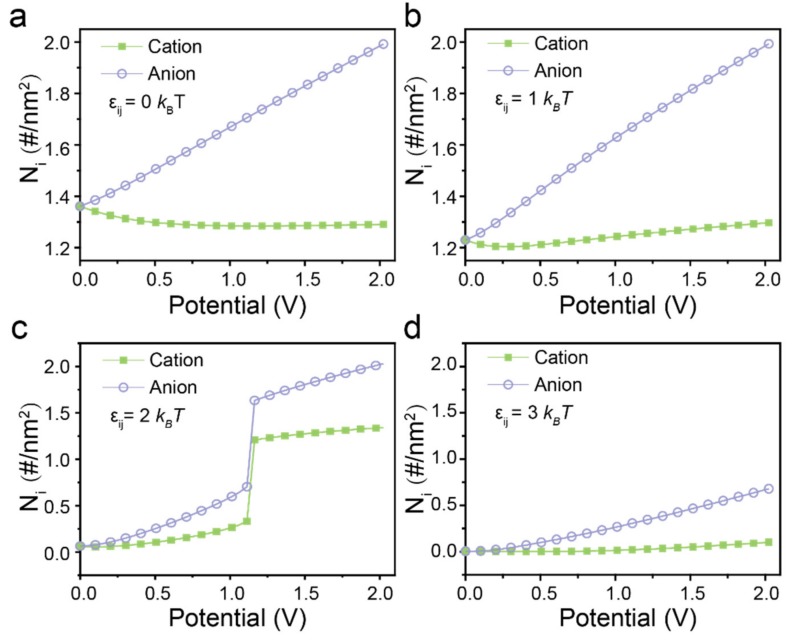
Number of ions inside nanopore versus applied electrical potential of RTILs with different dispersion force at $0\;k_{B}T$ (**a**), $1\;k_{B}T$ (**b**), $2\;k_{B}T$ (**c**) and $\;3\;k_{B}T$ (**d**), respectively.

To understand the *Q* – ψ curves and the anomaly phenomenon above, we investigated the average number density of cations and anions of RTILs with different dispersion forces inside the nanopores during the charging process. As shown in Figure 2a, the charging process for RTILs without regard to dispersion force could be divided into two stages. When at lower surface potential, similar variations of co-ions (cations) and counter-ions (anions) densities demonstrate that the dominant charging mechanism is the swapping of co-ions in the pore with the counter-ions from the bulk. In the next stage, few co-ions flow out from the pore as potential further raised which is indicated by the constant value of cation number density. The charging process is dominated only by the counter-ions insertion because of the long-range electrostatic correlations between the counter-ions and the charged pore electrodes. The charging process for RTILs with weak dispersion force is similar to that of the electrolyte without regard to dispersion force. However, the density of co-ions has a slightly increase at higher potential since more co-ions insert into the silt pore due to the attracted dispersion force between co-ions and counter-ions (Figure 2b). Moreover, the number density of ions is almost zero at low potential when the $\varepsilon_{ij}$ is elevated larger than $2\;k_{B}T$, which suggests that few ions could be in contact with the neutral slit pore because of the strong dispersion force between ions in the bulk (Figure 2c,d). Similar results have been observed previously in both experimental and simulation methods studies. For instance, Gogotsi et al. [26] found that 1-ethyl-3-methylimidazolium bis(trifluoromethylsulfonyl) imide ([EMIM][TFSI]) cannot wet micropores smaller than 0.75 nm at zero applied potential. From MD simulations, scientists also found that there exists a critical pore widthin wetting the neutral pore [35]. The critical pore width is shown dependent with the properties of the electrolyte [36], thus the capillary evaporation of ionic liquids with larger dispersion force may happen in the given silt pore. It should be noted that the ions density discontinuously jumps to a larger value at 1.16 V in the case of $\varepsilon_{ij}=2\;k_{B}T$, indicating a first-order phase transition [36]. The phase transition can be understood as the balance between the electrostatic correlations and the volume exclusion interaction. When a surface potential applied, the counter-ions start to accumulate in the pore resulting in electrowetting because of the electrostatic correlations.

**Figure 3:**
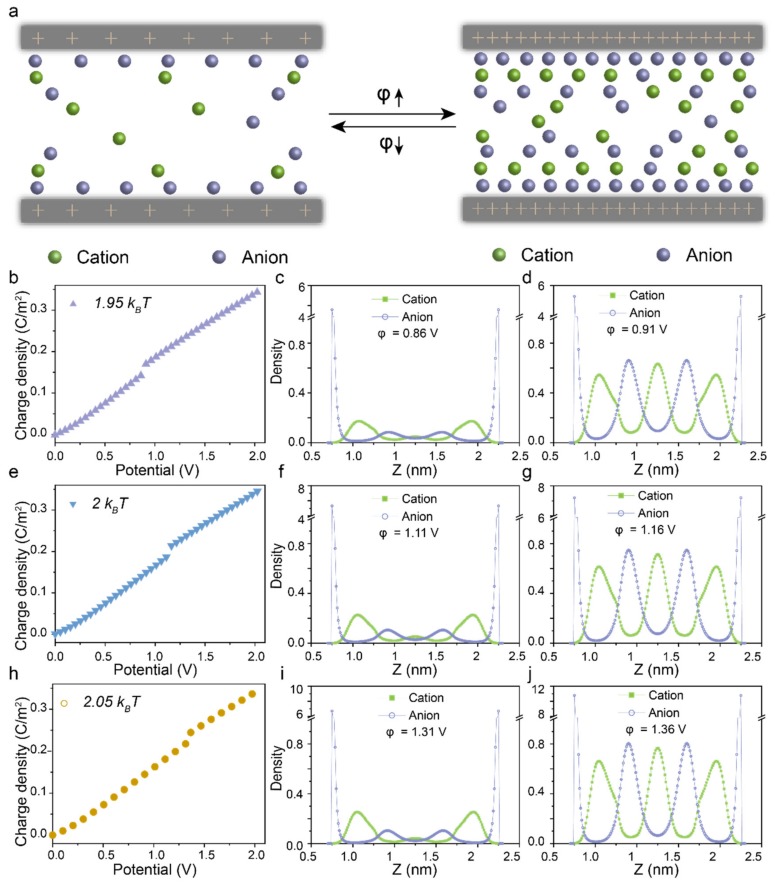
(**a**) Schematic of the voltage dependent phase transition. Theoretical prediction for the surface charge density versus the electrical potential of RTILs with different dispersion force at $1.95\;k_{B}T$ (**b**),$\;2\;k_{B}T$ (**e**) and $2.05\;k_{B}T$ (**h**). Density profiles of cations and anions inside the nanopore with a diameter of 2.5 nm at an electrical potential slightly below the phase transition potential at $1.95\;k_{B}T$ (**c**),$\;2\;k_{B}T$ (**f**) and $2.05\;k_{B}T$ (**i**). Density profiles of cations and anions inside the nanopore with a diameter of 2.5 nm at an electrical potential slightly above the phase transition potential at $1.95\;k_{B}T$ (**d**),$\;2\;k_{B}T$ (**g**) and $2.05\;k_{B}T$ (**j**).

Now we proceed to investigate the wettability of the model ionic system with variety dispersion force at $\varepsilon_{ij}=1.95\;k_{B}T,\;\;2\;k_{B}T$, and $2.05\;k_{B}T$ in charged pores (Figure 3a). As shown in Figure 3b,e,h, the surface potential corresponding to the first-order phase transition is positive related to the dispersion force between ions. Additionally, the density profiles of ions inside the pore under the surface potentials slightly below (Figure 3c,f,i) and above (Figure 3d,g,j) that according to the step change in the surface charge density are all formed of multiple ionic layers due to the over screening and the ionic excluded volume effects [44]. When at a slightly lower surface potential, the ions accumulating inside the nanopore with low-density manifest so that the fluid is in a vapor-like state. Few ions insert into the silt pore because of the strong volume exclusion effects and the dispersion force of ions in bulk. In the case of a slightly higher surface potential, the fluid is in an ionic liquid-like state indicated by the high density of ions inside the nanopore. At the pore center, there are more co-ions than the counter-ions, since the local charge inversion such as the local electrical potential has a sigh opposite to that of the surface charge.

**Figure 4:**
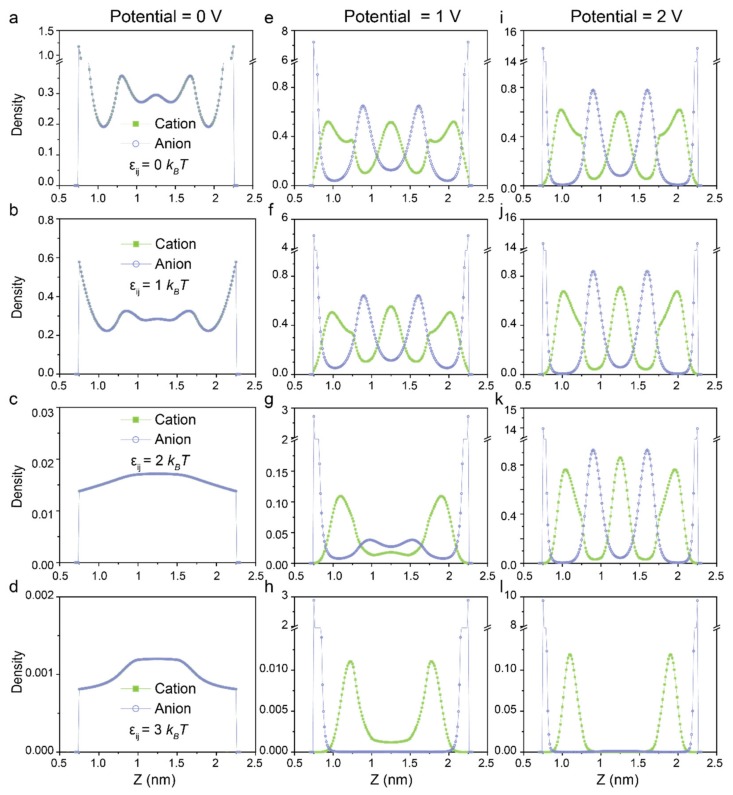
Density profiles of cations and anions with different dispersion force at $0\;k_{B}T$ (**a**, **e** and **i**),$\;1\;k_{B}T$ (**b, f** and **j**), $2\;k_{B}T$ (**c**, **g** and **k**) and$\;3\;k_{B}T$ (**d**, **h** and **l**) in a silt pore with diameter of 2.5 nm at electrical potential of 0 V (left), 1V (middle) and 2 V (right).

To further elucidate how dispersion force effects on the properties of surface charge density, we show in Figure 4 the local ionic density profiles of counter-ions and co-ions inside the nanopore. When the surface potential is set as 0 V, the densities of counter-ions and co-ions exhibit a homogeneity because cations and anions have the same ion diameter and valence. When the dispersion force is fixed at $\varepsilon_{ij}=0\;k_{B}T$ and $1\;k_{B}T$, the ion distributions versus z present the same pattern but with slight differences in the values (Figure 4a,b). By contrast, few ions insert into the silt pore due to the stronger dispersion force between ions in the bulk (Figure 4c,d). In the case of higher potential, the densities of counter-ions increase evidently with the enhanced exclusion of co-ions from the slit pore for RTILs without or with weaker dispersion force. In addition, the formation of a counterion layer near the electrode overcompensates the surface charge, leading to charge inversion and oscillatory ionic distribution (Figure 4e,f). As for RTILs with stronger dispersion force at $\varepsilon_{ij}=2\;k_{B}T$ and $\varepsilon_{ij}=3\;k_{B}T$, the counter-ions accumulate inside the silt pore by the electrostatic correlations, which in turn induce the co-ions owing to the strong dispersion force between opposite charged ions. However, the density of ions especially in the central of the pore is obviously less than that without regard to dispersion force (Figure 4g,h). It suggests that the ionic fluid exists in vapor-state inside the silt pore under these conditions. As the surface potential further raised, more counter-ions inserted into the nanopore so that the layer by layer distribution of counter-ions and co-ions become more evident at $\varepsilon_{ij}=0\;k_{B}T$ and $\varepsilon_{ij}=1\;k_{B}T$ (Figure 4i,j). It is worth noting that the multiple ionic layers emerge when fixed at $\varepsilon_{ij}=2\;k_{B}T,\;\;\psi =2\;V$, signaling the occurrence of the phase transition as the densities of counter-ions and co-ions are remote from the bulk density (Figure 4k). Whereas in the case of $\varepsilon_{ij}=3\;k_{B}T,\;\;\psi =2\;V$, the alternating layers do not show up since the electrostatic correlation is not strong enough to overcome the effects of the volume exclusion and dispersion force of ions in bulk (Figure 4l).

The differential capacitance $C_{d}$ used as a key quantity of practical interest for energy storage is defined as a derivative of the surface charge density with respect to the electrode potential:

$$C_{d}\;=\;\frac{\partial Q}{\partial \psi }$$

**Figure 5:**
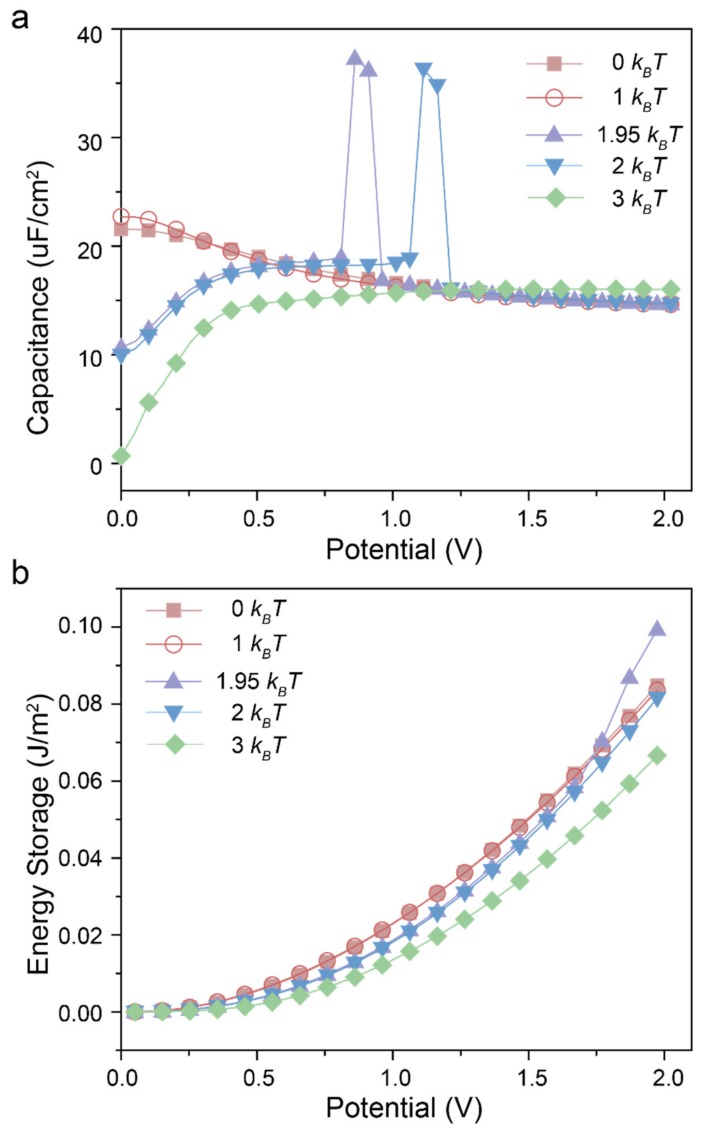
Differential capacitance (**a**) and energy storage (**b**) versus applied potential of RTILs with different dispersion force at $0\;k_{B}T$, $1\;k_{B}T$, $1.95\;k_{B}T$, $2\;k_{B}T$ and $3\;k_{B}T$, respectively.

Figure 5a presents the capacitance as a function of applied electrical potential of RTILs with different dispersion force. The $C_{d}-\psi$ curves exhibit a symmetric “bell shape” for RTILs without or with weaker dispersion force. As stated in Figure 2a, the near-symmetric densities of co-ions and counter-ions show that the main charging progress is the exchange of co-ions with the counter-ions from the bulk when applied low potential. Henceforth the differential capacitance decreases sharply due to the quick saturation of ion adsorption at a small range of electrical potential (here below 1.5 V). In the case of high potential (here above 1.5 V), the insertion of counter-ions dominates the charging process as indicated in Figure 2, leading to a slow decline of the differential capacitance.

When the dispersion force is large enough (e.g., $\varepsilon_{ij}=3\;k_{B}T$), the $C_{d}-\psi$ curve exhibits a “camel shape” instead of a “bell shape”, which is close to the experimental results [34]. When a surface potential is applied, both of counter-ions and co-ions flow into the nanopore, leading to a rapid increase of the surface charge density and capacitance since few ions exist in the slit pore at the initial stage due to the capillary evaporation. The differential capacitance increases to a maximum and then decreased because of the saturation of the surface charge. It’s worth mentioning that the capacitance discontinuity jumps to a finite value at the potential corresponding to the first-order phase transition when $\varepsilon_{ij}$ is set near $2\;k_{B}T$. The first-order phase transition occurred because the strong electrostatic correlations under high surface potential overcome the volume exclusion and dispersion force between ions in bulk. Therefore, a large number of counter-ions insert into the silt pore and in the meantime attract few co-ions due to the dispersion force between the opposite charged ions. In the latter case, the differential capacitance sharply declines to a stable value due to the saturation of surface charge. It is similar to the charging progress of RTILs with weaker dispersion force presented above.

The energy density in the slit pore could be calculated as below:

$$E=\int_{0}^{\psi_{0}}C_{d}V\;dV$$

where $C_{d}$ is the differential capacitance and the $\psi_{0}$ corresponds to one-half of the operation potential window (OPW). As shown in Figure 5b, the RTILs with larger dispersion force yield the lower energy density under most condition. However, the trend is changed under high potential (here is 1.82 V) for $\varepsilon_{ij}=1.95\;k_{B}T$ owing to the first-order phase transition. Unfortunately, there are no similar phenomena in the range of operation potential window when $\varepsilon_{ij}=2\;k_{B}T\;and\;3\;k_{B}T$. The small change in the dispersion force can significantly impact the capacitive performance and hence the energy density of EDL capacitors, which may inspire future experimental and computational efforts towards these directions.

## 4. Conclusions

In summary, we have investigated the effects of dispersion force between cations and anions of RTILs on the charging mechanism and the capacitive performance of electrical double layer (EDL) capacitors. The charging process of RTILs with weaker dispersion force is similar to that for electrolytes without dispersion force. At lower potential, the dominant charging mechanism is the swapping of co-ions by counter-ions inside nanopore. In the case of higher potential, the insertion of counter-ions plays a critical role in the charging progress. In the meantime, few co-ions also flow into the nanopore due to the dispersion force of opposite charged ions. Consequently, the capacitance exhibits a symmetric “bell shape”. As for RTILs with strong dispersion force, the insertion of both counter-ions and co-ions dominates the charging progress. In the initial stage, the surface charge density increases rapidly since few ions are inside the slit pore due to the capillary evaporation. As the potential is further raised, the surface charge density become saturated, leading to a decline of capacitance. Thus, the capacitance curves present a “camel shape”. Intriguingly, the discontinuous jump in the surface charge density demonstrates a structure transition when $\varepsilon_{ij}$ is set near $2\;k_{B}T$. In addition, the phase transition is voltage dependent, which occurs when the effects of electrostatic correlation overcome the volume exclusion and dispersion force between ions in bulk. Therefore, the capacitance jumps to a maximum as a large amount of ions flow into the silt pore, leading to the high energy storage. Despite much effort devoted into the optimization of ionic mixtures and selection of solvents, only limited attention has been given to the influence of dispersion force. We conclude by noting the small change in the dispersion force can significantly impact the charge density and capacitive performance of EDL capacitors, which may inspire future experimental and computational efforts towards these directions. Based on the above results, one could expect to improve the energy storage of supercapacitors through the manipulation of thermodynamic response by tailoring the chemical details of ions. For example, by adding/removing certain small groups such as methyl without significantly influencing other physical properties, the dispersion force between ions can be manipulated into certain values to enhance the energy density.

## References

[1] Chmiola J., Yushin G., Gogotsi Y., Portet C., Simon P., Taberna P.L. (2006). Anomalous increase in carbon capacitance at pore sizes less than 1 nanometer. Science.

[2] Ali B.A., Allam N.K. (2019). First-principles roadmap and limits to design efficient supercapacitor electrode materials. Phys. Chem. Chem. Phys..

[3] Burt R., Birkett G., Zhao X.S. (2014). A review of molecular modelling of electric double layer capacitors. Phys. Chem. Chem. Phys..

[4] Zhong C., Deng Y., Hu W., Qiao J., Zhang L., Zhang J. (2015). A review of electrolyte materials and compositions for electrochemical supercapacitors. Chem. Soc. Rev..

[5] Jiang D.E., Wu J. (2013). Microscopic insights into the electrochemical behavior of nonaqueous electrolytes in electric double-layer capacitors. J. Phys. Chem. Lett..

[6] Shao Y., El-Kady M.F., Wang L.J., Zhang Q., Li Y., Wang H., Mousavi M.F., Kaner R.B. (2015). Graphene-based materials for flexible supercapacitors. Chem. Soc. Rev..

[7] Fedorov M.V., Kornyshev A.A. (2014). Ionic liquids at electrified interfaces. Chem. Rev..

[8] Forse A.C., Merlet C., Griffin J.M., Grey C.P. (2016). New perspectives on the charging mechanisms of supercapacitors. J. Am. Chem. Soc..

[9] Largeot C., Portet C., Chmiola J., Taberna P.L., Gogotsi Y., Simon P. (2008). Relation between the ion size and pore size for an electric double-layer capacitor. J. Am. Chem. Soc..

[10] Jiang D.E., Jin Z., Henderson D., Wu J. (2012). Solvent effect on the pore-size dependence of an organic electrolyte supercapacito. J. Phys. Chem. Lett..

[11] Kondrat S., Perez C.R., Presser V., Gogotsi Y., Kornyshev A.A. (2012). Effect of pore size and its dispersity on the energy storage in nanoporous supercapacitors. Energy Environ. Sci..

[12] Seddon K.R. (1997). Ionic liquids for clean technology. J. Chem. Technol. Biotechnol..

[13] Simon P., Gogotsi Y. (2013). Capacitive energy storage in nanostructured carbon–electrolyte systems. Acc. Chem. Res..

[14] Dong K., Liu X., Dong H., Zhang X., Zhang S. (2017). Multiscale studies on ionic liquids. Chem. Rev..

[15] Lian C., Liu H., Li C., Wu J. (2019). Hunting ionic liquids with large electrochemical potential windows. AIChE J..

[16] Wang H., Xu Z., Kohandehghan A., Li Z., Cui K., Tan X., Stephenson T.J., King’ondu C.K., Holt C.M.B., Olsen B.C. (2013). Interconnected carbon nanosheets derived from hemp for ultrafast supercapacitors with high energy. ACS Nano.

[17] Yoo J.J., Balakrishnan K., Huang J., Meunier V., Sumpter B.G., Srivastava A., Conway M., Reddy A.L., Yu J., Vajtai R. (2011). Ultrathin planar graphene supercapacitors. Nano Lett..

[18] Zhu Y., Murali S., Stoller M.D., Ganesh K.J., Cai W., Ferreira P.J., Pirkle A., Wallace R.M., Cychosz K.A., Thommes M. (2011). Carbon-based supercapacitors produced by activation of grapheme. Science.

[19] Yang X., Cheng C., Wang Y., Qiu L., Li D. (2013). Liquid-mediated dense integration of graphene materials for compact capacitive energy storage. Science.

[20] Lukatskaya M.R., Mashtalir O., Ren C.E., Dall’Agnese Y., Rozier P., Taberna P.L., Naguib M., Simon P., Barsoum M.W., Gogotsi Y. (2013). Cation intercalation and high volumetric capacitance of two-dimensional titanium carbide. Science.

[21] Feng G., Zhang J.S., Qiao R. (2009). Microstructure and capacitance of the electrical double layers at the interface of ionic liquids and planar electrodes. J. Phys. Chem. C.

[22] Wu P., Huang J., Meunier V., Sumpter B.G., Qiao R. (2011). Complex capacitance scaling in ionic liquids-filled nanopores. ACS Nano.

[23] Kondrat S., Georgi N., Fedorov M.V., Kornyshev A.A. (2011). A superionic state in nano-porous double-layer capacitors: Insights from Monte Carlo simulations. Phys. Chem. Chem. Phys..

[24] Wang G., Zhang L., Zhang J. (2012). A review of electrode materials for electrochemical supercapacitors. Chem. Soc. Rev..

[25] Liu R., Duay J., Lee S.B. (2011). Heterogeneous nanostructured electrode materials for electrochemical energy storage. Chem. Commun..

[26] Vatamanu J., Hu Z., Bedrov D., Perez C., Gogotsi Y. (2013). Increasing energy storage in electrochemical capacitors with ionic liquid electrolytes and nanostructured carbon electrodes. J. Phys. Chem. Lett..

[27] Hayes R., Warr G.G., Atkin R. (2015). Structure and nanostructure in ionic liquids. Chem. Rev..

[28] Wang Y., Wang C., Zhang Y., Huo F., He H., Zhang S. (2019). Molecular insights into the regulatable interfacial property and flow behavior of confined ionic liquids in graphene nanochannels. Small.

[29] Tong J., Xiao X., Liang X., von Solms N., Huo F., He H., Zhang S. (2019). Insights into the solvation and dynamic behaviors of a lithium salt in organic- and ionic liquid-based electrolytes. Phys. Chem. Chem. Phys..

[30] Trulsson M., Algotsson J., Forsman J., Woodward C.E. (2010). Differential capacitance of room temperature Ionic liquids: The role of dispersion forces. J. Phys. Chem. Lett..

[31] Forsman J., Woodward C.E., Trulsson M. (2011). A classical density functional theory of ionic liquids. J. Phys. Chem. B.

[32] Alam M.T., Islam M.M., Okajima T., Ohsaka T. (2007). Measurements of differential capacitance at mercury/room-temperature ionic liquids interfaces. J. Phys. Chem. C.

[33] Alam M.T., Islam M.M., Okajima T., Ohsaka T. (2008). Capacitance measurements in a series of room-temperature ionic liquids at glassy carbon and gold electrode interfaces. J. Phys. Chem. C.

[34] Lockett V., Sedev R., Ralston J., Horne M., Rodopoulos T. (2008). Differential capacitance of the electrical Double layer in imidazolium-based ionic liquids:  Influence of potential, cation size, and temperature. J. Phys. Chem. C.

[35] Shrivastav G., Remsing R.C., Kashyap H.K. (2018). Capillary evaporation of the ionic liquid [EMIM][BF4] in nanoscale solvophobic confinement. J. Chem. Phys..

[36] Liu K., Zhang P., Wu J. (2018). Does capillary evaporation limit the accessibility of nonaqueous electrolytes to the ultrasmall pores of carbon electrodes?. J. Chem. Phys..

[37] Szparaga R., Woodward C.E., Forsman J. (2012). Theoretical prediction of the capacitance of ionic liquid films. J. Phys. Chem. C.

[38] Szparaga R., Woodward C.E., Forsman J. (2013). Capillary condensation of ionic liquid solutions in porous electrodes. J. Phys. Chem. C.

[39] Lian C., Liu K., Liu H., Wu J. (2017). Impurity effects on charging mechanism and energy storage of nanoporous supercapacitors. J. Phys. Chem. C.

[40] Jiang D.E., Jin Z., Wu J.Z. (2011). Oscillation of capacitance inside nanopores. Nano Lett..

[41] Lian C., Jiang D.E., Liu H., Wu J.A. (2016). Generic model for electric double layers in porous electrodes. J. Phys. Chem. C.

[42] Jiang D.E., Wu J. (2014). Unusual effects of solvent polarity on capacitance for organic electrolytes in a nanoporous electrode. Nanoscale.

[43] Wu J., Li Z. (2007). Density-functional theory for complex fluids. Annu. Rev. Phys. Chem..

[44] Wu J., Jiang T., Jiang D.E., Jin Z., Henderson D. (2011). A classical density functional theory for interfacial layering of ionic liquids. Soft Matter.

[45] Lian C., Liu K., Van Aken K.L., Gogotsi Y., Wesolowski D.J., Liu H.L., Jiang D.E., Wu J.Z. (2016). Enhancing the capacitive performance of electric double-layer capacitors with ionic liquid mixtures. ACS Energy Lett..

[46] Boda D., Fawcett W.R., Henderson D., Sokołowski S. (2002). Monte Carlo, density functional theory, and Poisson-Boltzmann theory study of the structure of an electrolyte near an electrode. J. Chem. Phys..

[47] Gillespie D., Valiskó M., Boda D. (2005). Density functional theory of the electrical double layer: The RFD functional. J. Phys. Condens. Matter.

[48] Yu Y.X., Wu J., Gao G.H. (2004). Density-functional theory of spherical electric double layers and zeta potentials of colloidal particles in restricted-primitive-model electrolyte solutions. J. Chem. Phys..

[49] Li Z., Wu J. (2006). Density functional theory for planar electric double layers:  Closing the gap between simple and polyelectrolytes. J. Phys. Chem. B.

[50] Lian C., Zhao S., Liu H., Wu J. (2016). Time-dependent density functional theory for the charging kinetics of electric double layer containing room-temperature ionic liquids. J. Chem. Phys..

[51] Yu Y.X., Wu J.Z. (2002). Structures of hard-sphere fluids from a modified fundamental-measure theory. J. Chem. Phys..

[52] Fouad W.A., Haghmoradi A., Wang L., Bansal A., Hammadi A., Asthagiri A., Asthagiri D., Djamali E., Cox K.R., Chapman W.G. (2016). Extensions of the SAFT model for complex association in the bulk and interface. Fluid Phase Equilib..

[53] Lian C., Kong X., Liu H., Wu J. (2016). On the hydrophilicity of electrodes for capacitive energy extraction. J. Phys. Condens. Matter.

